# Cardiology and Neurophysiological Stimulation of Internet Gaming Disorders: A Systematic Review

**DOI:** 10.2174/011573403X295560240530104352

**Published:** 2024-06-21

**Authors:** Immaculate Joy Selvam

**Affiliations:** 1 Department of Electronics and Communication Engineering, Saveetha Engineering College, Thandalam, Chennai, India

**Keywords:** Internet gaming disorder, DSM-5, sympathetic activity, greater thalamic activation, neuroimaging, cognitive assessments

## Abstract

**Background:**

Internet Gaming Disorder (IGD) is recognized as a mental health condition associated with excessive video gaming, leading to functional impairments. The inclusion of IGD in the DSM-5 has underscored the importance of comprehensively understanding its physiological and psychological effects.

**Objective:**

This systematic review aims to analyze and synthesize existing literature on the cardiophysiological and neurophysiological activities of individuals diagnosed with IGD, with a focus on identifying patterns, trends, and implications for clinical practice and future research.

**Methods:**

A systematic search was conducted in PubMed and Scopus databases to identify relevant studies published up to 2023. The search strategy included terms related to IGD, cardiophysiology, neurophysiology, and relevant measurement techniques. Inclusion criteria encompassed peer-reviewed research articles and clinical trials examining cardiophysiological (*e.g.*, heart rate variability, blood pressure) and neurophysiological (*e.g.*, brain imaging, electroencephalography) parameters in individuals with IGD. Exclusion criteria were applied to ensure methodological rigor and relevance to the research question.

**Results:**

The initial search yielded 1320 papers related to IGD, of which twenty studies met the eligibility criteria and were included in the review. Data extraction and synthesis focused on key cardiophysiological and neurophysiological outcomes observed in individuals with IGD compared to healthy controls. Findings revealed decreased Heart Rate Variability (HRV), increased sympathetic activity, and executive control deficits in IGD individuals based on Electrocardiogram (ECG) recordings and cognitive assessments. Neuroimaging studies demonstrated heightened brain activation in the lateral and prefrontal cortex, altered reward processing, and impulse control mechanisms among IGD subjects. Gender-specific differences were noted, with males exhibiting distinct thalamic activation striatum and decreased Regional Homogeneity (ReHo) in the right Posterior Cingulate (rPCC) compared to females.

**Discussion:**

The synthesized evidence indicates a complex interplay between excessive gaming and cardiophysiological/neurophysiological changes, highlighting the need for multidimensional assessments in diagnosing and managing IGD. Implications for clinical practice include early detection using ECG, EEG, and advanced neuroimaging techniques, as well as personalized interventions tailored to individual characteristics and gender-specific differences.

**Conclusion:**

This systematic review provides a comprehensive overview of the cardiophysiological and neurophysiological activities associated with Internet Gaming Disorder. The findings underscore the need for further research to elucidate underlying mechanisms, develop standardized diagnostic protocols, and optimize targeted interventions for individuals with IGD.

## 1 INTRODUCTION

In the past two decades, the necessity of the internet and mobile phones has increased drastically and taken up a major role in leisure activities [1]. A survey shows that in the USA, children around 8-10 years old spend 8 hours a day, and adolescents spend more than 11 hours per day using electronic gadgets [2]. Video gaming seems to be a stimulating activity for most young individuals [3]. However, the negative impact is linked to the increasing usage of digital technologies and their related disorders like internet gaming disorder (IGD) [4- 6]. In India, many studies have reported the addiction of young adults to gaming and generating revenue over internet games [7]. The consequent increase in IGD impacts severe risk factors such as physiological disorders and mental health [8-11]. The research on IGD started in the early 1970s after the release of the first video game [12]. Meanwhile, DSM-5 included IGD before being recognized as a mental health disorder and warranted further investigation [13, 14]. The international consensus assessed IGD using the new DSM-5 approach and lists 9 criteria for IGD: (A) pre-occupation, (B) withdrawal, (C) tolerance, (D) reduce/stop, (E) give-up other activities, (F) continue despite problems, (G) deceive/ cover up, (H) escape adverse mood, and (I) risk/ loss of relationship opportunities [15]. Numerous studies employed various questionnaires to classify IGD with inconsistent criteria, and thus, DSM-5 classification of IGD is an important aspect [16-18]. Criteria E is not assessed in the beard diagnostic questionnaire [19]. Criteria F was not assessed in the young’s internet Addiction Test (IAT) and the Young Diagnostic Questionnaire (YDQ) [20-22]. Criterion E, G, or I is not assessed in the scale for the assessment of the internet and computer game addiction-gaming module (AICA-S-gaming) [23].

The adverse effect of intermittent use of the Internet has become a collective issue [24]. Specifically, continuous use of internet games is now regarded as an addiction-related mental disorder, which is closely associated with physiological, psychological, and social adjustment problems [25, 26]. The psychological and physiological information can be delivered by emotion, and thus, the IGD addicts, the characteristics of emotion as well as the Autonomic Nervous System (ANS) are best illustrated in many studies [27]. Heart Rate Variability (HRV) serves as a file of guidelines involving parasympathetic and sympathetic activity over ANS[28]. HRV is the most commonly used index for cardiovascular risk stratification and an important mirroring agent of heart rate fluctuations. These emotions are the reaction of persuasive factors in subjects with IGD [29]. There are several evidence to indicate that prefrontal neural functions and executive control are related to vagally mediated HRV (High-frequency HRV) [30]. Vagally mediated HRV is reduced to a greater extent during mental tasks that are linked to inefficient prefrontal control over cognitive performance [31]. The individuals show difficulties when engaged in online gaming, and thus, vagally mediated HRV is suppressed greatly with the severity of IGD and Gray Matter Volumes (GMVs) of the prefrontal cortex [32]. The accurate assessment of ANS function can be obtained when HRV response is measured at particular stimuli as well as in the rest state, thus, there will be better reflections in ANS responses for the changing environment [33].

The most important insight of IGD is the brain's reaction during gaming-related cues. The IGD subjects have upgraded longing for and weakened authority, leading to inclinations over liquor and chronic drug habits [34]. IGD people show subjective inclinations towards gaming-related boosts at higher rates and more noteworthy sign-initiated actuation in the striatum when presented with gaming-related pictures [35, 36]. The fundamental trigger to addiction is needed likewise, subjects with IGD have actuation of PFC when presented with gaming related and activation of the dorsal and ventral striatum when exposed to gaming-related pictures [37]. IGD and Substance Use Disorder (SUD) have similar neurobiological mechanisms and clinical features such as withdrawal symptoms, exercise behaviors, and cravings [38, 39]. To explain the underlying neural mechanisms in a neurologic perspective, functional Magnetic Resonance Imaging (fMRI) has been used in recent research [40]. IGD subjects also show greater activation in the center occipital gyrus, second-rate parietal lobules, and Dorsolateral Prefrontal Cortex (DLPFC) [41]. The Regional Homogeneity (ReHo) method has been involved in evaluating regional activation patterns through indices of localized concordance [42]. Without earlier information on the structure and capacity of the mind, ReHo finds the territorial synchronization of the entire cerebrum levels, which may contrast with the sex bunches [43].

### 1.1 Objective

The prime goal of this study is to review the scientific literature to elicit the impact of IGD on the physiological functions of the heart and brain. The review summarizes the methods and conclusions of major studies that were carried out to reveal the psychological and physiological changes associated with individuals with IGD.

## 2 MATERIALS AND METHODS

In view of the underlying criteria, the current review is directed to 1) surveying neurological and cardiological components in IGD, 2) utilizing neuroimaging methods, 3) writing in English, and 4) recent distributions in peer-evaluated journals. Studies were looked at from the PubMed database. The pursuit included most regular kinds of neuroimaging procedures, for example, Electroencephalogram (EEG), practical, attractive reverberation imaging, loosen state utilitarian attractive reverberation imaging (rfMRI), and Voxel-Based Morphometry (VBM) and Electrocardiogram (ECG) to record heart signals. Other inquiry systems incorporate IGD, ECG, HRV, EEG, disorder, bio-signals, and neuroimaging. The articles were looked at from Pubmed and Scopus databases. The search strings included “internet gaming disorder [or] internet gaming and heart disorder [or] internet gaming - ECG study [or] internet gaming - EEG study.” Thereafter, abstracts of suitable records with potential biomarkers of IGD were evaluated. Towards the end, there were 20 studies left with cardiological and neurophysiological with ECG and EEG, respectively that were alone taken for the study. Neither the type of language nor time bound on publication of articles was considered. Each paper's title and abstract were screened for qualification. In view of the techniques and methods included, relevant papers were recovered and reviewed, as depicted in Fig. (**1**).

## 3 RESULTS

### 3.1 IGD Evaluation Criteria based on ECG Studies

The Internet Gaming Disorder has been commonly diagnosed by DSM-5 criteria [44, 45]. The exclusion criteria for the subjects that are followed in the studies for the HRV analysis are subjects with significant psychiatric symptoms like depression, anxiety, and alcohol-related problems, childhood symptoms of attention-deficit/hyperactivity disorder, and impulsivity. Moreover, subjects with neurological disorders or medical illnesses and also cardiac disease or endocrine disease. The main reason behind these exclusion factors is that these parameters were found to affect the HRV value significantly. After the HRV recording ended, each participant completed several self-report questionnaires to assess comorbid psychiatric symptoms of IGD. All subjects completed the Beck Depression Inventory (BDI), the Beck Anxiety Inventory (BAI), the Alcohol Use Disorders Identification Test (AUDIT), and the Wender Utah Rating Scale (WURS) test to evaluate for childhood symptoms of Attention-Deficit/Hyperactivity Disorder (ADHD), and the Barratt Impulsiveness Scale, version 11 (BIS-11) to evaluate for impulsivity [46].

The parameters that are commonly discussed in most of the studies are Low Frequency (LF) which reflects parasympathetic and sympathetic nerve activity, whereas the High Frequency (HF) mainly represents the parasympathetic activity. The time domain index of HRV is SDNN (Standard Deviation of N-N intervals) that, indicates the overall HRV and the Root Mean Squared Difference of Successive N-N intervals (RMSSD) that indicates short-term changes in the Heart Rate which is used for predict the parasympathetic activity.

The persistent use of internet games may affect the heart rate of the individual, and there is a chance for significant changes in HRV parameters. Considering this scenario and the importance of IGD, various studies were analyzed to give a clear idea about the cons of internet gaming and its effects.

### 3.2 HRV Parameters Response during Gameplay

In a couple of studies, we found the subjects selected were only young males, and IGD was measured based on their IAT score (score above 50). The HRV was recorded before and after playing the internet game League of Legends. The difference in the analysis method is that high attention [47] and low attention HRV were recorded [48]. Both studies reported no critical difference between the IGD and healthy controls in terms of self-reporting, anxiety, depression, and alcohol-related problems. The results conclude that HRV response is specific to the gaming situation, and it varies based on addictive patterns of gaming, and this has an adverse effect on the executive control of the subject. There was no statistical difference in the HRV parameter between healthy controls and IGD subjects, but the latter had notable differences in RMSSD and lnHF values, which reflect vagal activity [49]. Meanwhile, the other studies [50] concluded that HRV response was altered during gameplay, and the severity of IGD has a significant correlation with the suppression of HF-HRV. Both studies found that subjects with IGD had a significant decrease in their HF-HRV component during game play when compared with healthy controls and baseline values.

In another study of 31 subjects, they were given a resting period of 5 minutes and analyzed biosignals recorded during and after playing the game “League of Legends.” The observation over the measuring device showed that computer gaming was shown to increase the sympathetic activity of the nerve, increased blood pressure, and SDNN during the game-playing state. Other than the HRV, no apparent differences were observed with the other biosignals. They also observed the rise in skin temperature during the game and its failure to reach back to the initial temperature [51].

Studies on cardiovascular diseases have shown evidence of high mortality throughout the world. This is found to be very common in most developing countries, accompanied by Sudden Cardiac Death (SCD). Compared to many of the invasive methods, HRV happens to be a potential non-invasive biomarker. The study by Sessa F, *et al.* has reported the prominent relationship between HRV and SCD with high- and low-risk cohorts. Though the risk of SCD increases with the age ratio of young people, it also contributes to a significant proportion of variations in heart rate due to stressful habits [52, 53]. The IGD subsidize a vital role among young children and adolescents with enormous amounts of stress that can give rise to heart rate fluctuations. This stress dwells in the rise of parasympathetic activity, leading to SCD among the younger generation. So, the increased gaming disorder may pave the way for increased risk of SCD.

The Inter-Beat Intervals (IBI) (or) RR Intervals were recorded for a period of 5-minute time duration while the participants were seated in a relaxed state with their eyes closed. With the obtained data, it was found that IGD patients had increased heart rates and decreased HRV. The lower HF value associated with IGD subjects when compared to controls is declared as an indication of lower regulatory function. Among the time domain parameters, decreased SDNNi had resulted in IGD patients. As there is a positive correlation between the heart rate and the Y-IAT score, it is said that elevated heart rate could be related to the urge to engage more with internet games and to a state of addiction [54].

### 3.3 Analysis of Significant HRV Features Due to internet addiction

Apart from HRV, some studies analyzed the emotions of both males and females under the category of (HIA) high-risk internet addiction and (LIA) low-risk internet addiction based on DSM-5 criteria with the Chen Internet Addiction Scale (CIAS) having a cut-off score of 63. The fundamental physiological signals, which included ECG, facial expression, and respiration, were recorded. Online game films were displayed, and after each film, the emotional intensity was ranked on a scale of 1 to 9. They declared that IGD had a weak physiological reaction and more positive emotions towards the online game, with the occurrence of higher valence values and low arousal values [55]. Hsieh. *et al.* observed significant differences among both the IA groups during the emotional induction states in their study. It was evident that, both before and after the induction of emotions (both positive and negative), the RSA (respiratory sinus arrhythmia) values of HIA were low when compared with LIA. During the time of negative emotion (anger or fear) by the subjects, there was a reduction in RSA values [56].

A study among 68 adolescent males notified as internet gaming addiction (IGA) and non-IGA groups exhibited significant differences mainly in parameters like SDNN and RMSSD of time domain as given in Table **1**. Moreover, log TP, log LF, and log HF of the frequency domain were found to have significant variations. Log TP and SDNN had remarkably low significance in the IGA group, indicating a rise in sympathetic activity and a fall in cardiac vagal tone. Moreover, the IGA group had higher log LF and lower log HF. IGA was correlated with minimal resting-state HRV levels, furthermore, overall HRV levels constituted by SDNN and log TP were correlated in a negative way with SI subscale scores and DS14 total. The results henceforth suggest that young adults with IGA show significant variations in HRV parameters and are classified as type D personnel traits [57].

The common observation from three of the considered studies is that excessive involvement in internet gaming developed serious emotional and neurobiological stress-related problems among young adults [58]. Therefore, from the obtained HRV data, it was found that the sympathetic nerves became more active (with the help of an apparent increase in VLF), and parasympathetic nerves were less active when compared to non-IGD subjects [59-61]. The affected young adults due to addiction akin to internet gaming have been assessed in terms of many biological parameters. In one of the papers, the main factor of consideration was the stress level of the subjects during the gameplay. The samples, such as hair and saliva, were considered for analysis purposes, and the stress response of subjects was measured using a series of tests. Finally, it was concluded that patients with IGD were observed with greater symptoms of depression and greater levels of anxiety [62]. In Summary, subjects with IGD were found to have abrupt changes in the heart rate, blood pressure, sympathetic activity, and many other parameters.

## 4 DISCUSSION ON IGD IMPACT THROUGH EEG STUDIES

Neuroimaging in IGD is one of the fast-growing fields of research. Due to gameplay, IGD subjects show different changes in their neural regions. For the analysis of IGD using neuroimaging, different parameters were observed like fMRI, rsfMRI (Resting-State Functional Magnetic Resonance Imaging), Voxel, and EEG (Electroencephalogram). Studies involving various subjects have been consolidated for this review: i) gender-related IGD differences [63-66] ii) The analysis of EEG between the RGU and IGD has been related in the papers [36, 49] iii) IGD has also been analyzed in correlation with comorbid depression subjects [67] iv) EEG analysis between the healthy controls and IGD [68-70] v) IGD analysis between pro-gamers [71]. Exclusion of subjects with (1) previous hospitalization for psychiatric disorders such as anxiety, depression, or attention-deficit hyperactivity disorder, (2) substance use disorders, (3) mental retardation (4) neurological illness or injury and (5) intolerance to MRI [72]. Study findings by Liu J. *et al*., have proved that a significant change in brain due to internet addiction disorder. The Regional Homogeneity (ReHo) method used in the study was conducted over the young adolescent people with fMRI who had observed encephalic characteristic changes. The abnormal brain functions with increased voxels were noted in the IGD group compared to the controls [73].

The diagnostic criteria for IGD persons scored above 50 in YIAT (Young’s Internet Addiction Test) and met at least 5 points out of 9 in DSM-5 criteria. The subjects who did not satisfy these criteria were said to be HC (healthy controls). These healthy control subjects are chosen to be right-handed and with normal vision [74, 75]. Additional diagnostic criteria included i) Assessment about structured psychiatric interviews (MINI) [33, 36, 48] ii) Modified Internet addiction test, that is Young’s Brain Imaging and Behavior Diagnostic Questionnaire for Internet Addiction, YDQ)] modified by Beard (Beard and Wolf 2001) [37] iii) The severity of IGD was diagnosed by the Chen Internet Addiction Scale (CIAS) of 26 questionnaire carrying 4 marks and the cut off is 63/64 [37] iv) Beck Depression Inventory-II (BDI-II; Beck, Steer, Ball, & Ranieri, 1996), the Beck Anxiety Inventory (BAI; Beck, Epstein, Brown, & Steer, 1988) questionnaires and the Structured Clinical Interview for DSM-IV. The exclusion parameters for the subjects are (a) BDI score> 13, (b) BAI score > 15, (c) Axis I psychiatric disorders, and (d) head injury or history of trauma [50]. Subjects had undergone so many tests such as the Young Internet Addiction Scale (YIAS), Child Depressive Inventory (CDI), Beck Anxiety Inventory (BAI), Child Behavior Checklist (CBCL), and Korean ADHD Rating Scale (K-ARS) [52]. The game chosen for the diagnosing criteria was the online multiplayer game “League of Legends” and “World of Warcraft.”

The fMRI resting data were acquired using a 3T MRI system with respect to specific parameters such as TE, TR, FOV, thickness, and slices [76]. Significant interactions were observed with subjects at rest in their brain features based on sex groups such as the right Posterior Cingulate (rPCC), left Middle Ocipital gGyrus (lMOG), right Middle Temporal Gyrus (rMTG), and right Postcentral Gyrus (rPG) [36]. During the Post-hoc analysis, it was found that male IGD had decreased ReHo in the rPCC, which was negatively associated with their IAT scores, and increased ReHo in the other two parameters, IMOG and rMTG when compared to female IGD and same-sex RGU’s. Studies have revealed that brain features differ between the male and female sex groups [38], and sex-specific arrangements interrelate with their cerebral activities. The voxel-based signals were also analyzed. Moreover, the IGD subjects were identified as non-regulative in their negative emotions compared to those of recreational game users [77]. The analysis was conducted by the regional Amplitude of Low-Frequency Fluctuation (ALFF). For sex-diagnosis interaction on ALFF, statistical analyses were performed using a two-factor analysis of covariance (ANCOVA) using SPM8. The ALFF values in the orbital part of the left SFG (superior frontal gyrus) were lower in IGD males than in HC males. This factor was highly associated with higher impulsivity, and no similar difference was noted between IGD and HC females [38]. When the orbital part of the left SFG was set as the seed region, IGD males had lower seed connectivity with the right DLPFC (t dorsolateral prefrontal cortex), the right AG, and the PCC (posterior cingulate cortex) than HC males. In addition, IGD males showed lower connectivity between the orbital part of the left SFG and PCC compared to that of IGD males [37].

On the other hand, Dong *et al.* have concludedthat males are more vulnerable to IGD than females by comparing the RGU subjects. Prior to gaming, males had greater activation in the striatum, orbitofrontal cortex, inferior frontal cortex, and bilateral declive. During gaming, male subjects had greater activation in the medial frontal gyrus and bilateral middle temporal gyri. Post-and pre-analysis demonstrated that male subjects had greater thalamic activation than female subjects [33]. IGD, relative to RGU subjects, has shown higher craving scores for gaming cues before and after gaming. This was completely contrasted with the other papers on sex differences because they had conducted cue-related tasks. The imaging results provide further support for prefrontal–striatal interactions distinguishing groups with IGD and those with RGU, but there were no group differences in the right hemisphere lentiform-to-ACC pathway or the ACC-to-lentiform pathway [78].These gender differences should be taken into account for future studies and the treatment for IGD.

### 4.1 Influence of IGD in Brain Imaging Amidst Tasks

Many studies have been conducted in the literature with different tasks and different parameters. The game-promoting behavior was assessed through the gaming *versus* typing task for the subjects. Subjects with IGD had increased craving and brain activation of the lateral and prefrontal cortex, striatum, and precuneus during gaming-related stimuli in both pre- and post-gaming sessions. Individuals with RGU had no enhanced brain activity and suggests that gaming may promote more game cravings in IGD than RGU [36]. Similar brain activity (or abnormalities in the neural regions) was reported during the conduction of the addiction Stroop test over IGD candidates [35]. Adjoining the previous studies, a cue reactivity task was conducted on IGD-recovered subjects, who were reported to be sensitive to gaming cues. Brain activity in the lentiform nucleus and changes in self-reported cravings were observed [79].

A new study by Lee *et al.* employed IGD subjects with comorbid depression and without comorbid depression and healthy controls [31]. IGD subjects of both groups had stronger pgACC FC within the right precuneus, posterior cingulate cortex, and left inferior frontal gyrus/insula than the control group. In comparison with IGD without comorbid depression, the IGD subjects with comorbid depression had stronger dACC FC in the left precuneus and right cerebellar lobule [80, 81]. When a comparison was carried out between IGD and pro gamers, the IGD adolescents showed higher brain activity developed in the left orbitofrontal cortex, as in Fig. (**2**) [52]. When both brain imaging parameters and EEG signals were analyzed, the resting-state functional neural networks were inefficient and decreased theta functional connectivity in IGD when compared with HC [82]. In general, the negative impact of IGD adolescents on psychosocial health verified across the DSM-5 criteria [56, 57] coincides rightly with the current review.

A pair of studies were identified for related EEG signals for IGD analysis. The study evolved into the go/no-go task. Adolescents with IGD exhibited lower inhibitory control for no-go trials and more risk-seeking by choosing risky choices than the controls. The IGD adolescents showed decreased no-go P3 and blunted Feedback-Related Negativity (FRN) amplitudes (gain FRN) with no loss [55]. Another study has reported that IGD patients with low resilience had increased alpha coherence, especially in the right hemisphere. There were also indirect effects of IGD on alpha coherence in the right hemisphere, though depressive symptoms and stress levels were significant only in IGD patients with a lower degree of resilience in Table **2** [57].

## 5 DISCUSSION

All studies in the literature have found remarkable variation levels in both the kind of biomarkers (*i.e*) HRV and functional encephalic change in the brain. As previously stated HRV represents a quickly accessible assessment of the cardiac-based risk factors. It enumerates the autonomic cardiac innervations due to the increased stress of IGD, which may result in sudden cardiac death. Results of low HRV serve as an independent predictor of mortality in congestive heart failure patients. With respect to EEG studies, a significantly increased value of sReHo and dReHo is found with IGD samples compared to normal people. A causal relationship between abnormal brain activity and IGD could not be determined, as many were cross-sectional studies. Hence, a longitudinal study may be required to support the argument and findings in the future.

To date, prevention of sudden cardiac death remains a big social challenge as it requires balancing between both the risk and benefits of an individual. In this case, more multi-marker studies are required to be conducted in the future to improve the prediction of risk associated with IGD.

## CONCLUSION AND FUTURE DIRECTIONS

In most of the studies, we observed that DSM-5 had been used as diagnostic criteria, and the process involved with it is, moreover questionnaire-based. There is a chance that the subjects may provide incorrect answers or fail to provide accurate information. The high morbidity factor due to IGD encourages the researchers to find highly reliable and potential biomarkers. This can aid in early detection and timely treatment thereafter. To emphasize the impact of the usage of internet games, the awareness about the gaming disorder, and its adverse effect on the health of human beings, there is always a need for a proper diagnostic criterion for identifying individuals with IGD. Therefore, we suggest that the analysis methods that are used in the studies could be framed in such a way that they can be used as a diagnostic approach. We suggest that future research could be carried out, such that the HRV data obtained from the ECG of the individuals could be analyzed and any particular parameter like HF/LF component of the HRV could be defined and can be used as an indicator of IGD.

With the current knowledge, best efforts were taken in analyzing the studies in the literature, but it has a few limitations. The first reason is the limited number of researchers was found in studying IGD with respect to biomarkers. So, this raises the need to conduct practically applicable research with biomarkers to identify IGD. Secondly, there is no standardization of scales or protocols defined to diagnose IGD, as different studies have used different criteria for evaluation. Finally, most of the studies have considered male adolescents to study cardiology or neurophysiological factors that influence IGD. It is a known fact that both these factors differ with respect to different age groups and also between males and females.

## Figures and Tables

**Fig. (1):**
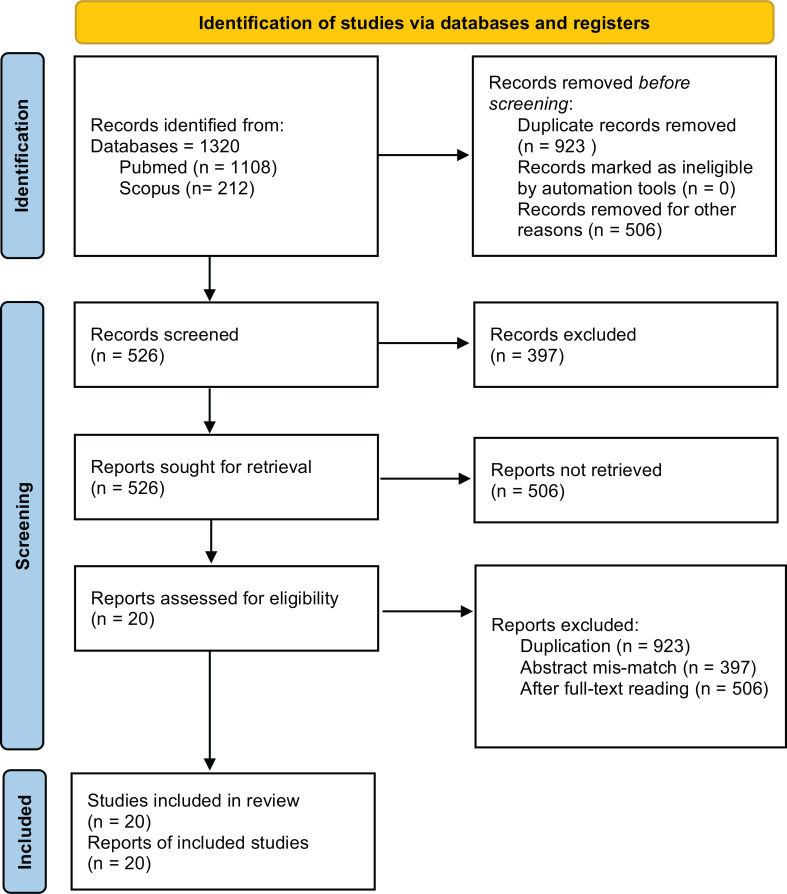
PRISMA Flow diagram of record search methodology.

**Fig. (2):**
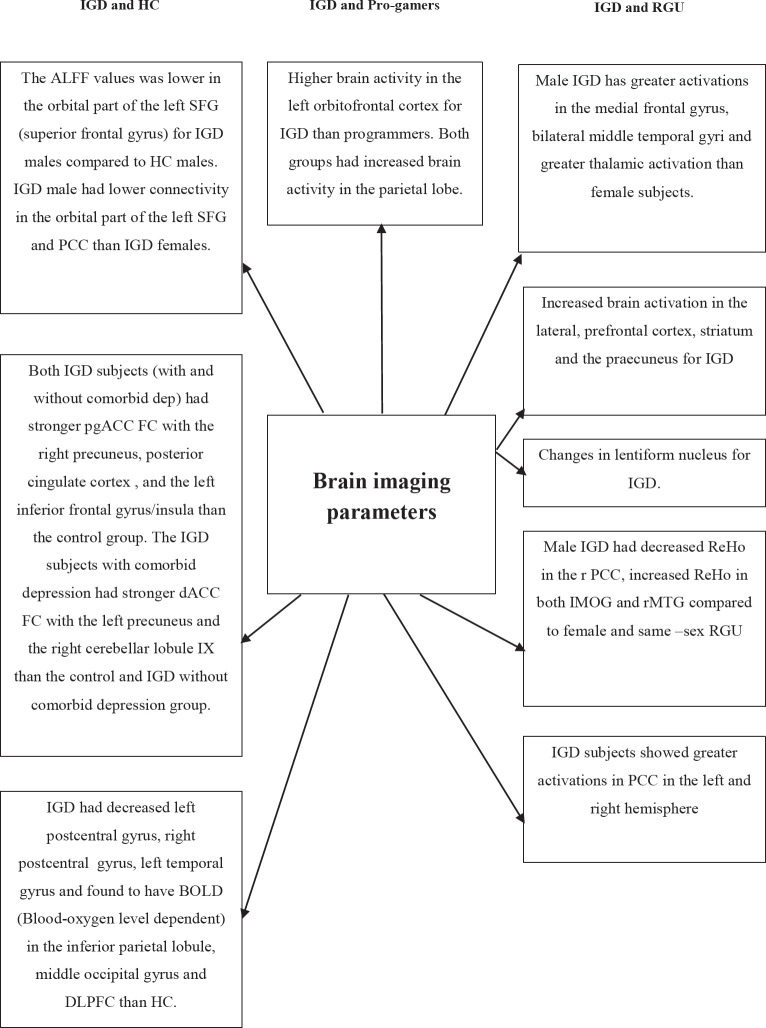
Comparison between IGD and pro-gamers on brain imaging parameters.

**Table 1:** Time domain HRV parameters with respect to IGD and their controls.

**Parameters**	**Controls**	**IGD**	**REF NO**
RMSSD	41.9 ± 17.3	35.2 ± 11.8	[12]
	48.42 ± 31.99	23.78 ± 11.34	[4]
	46.16 ± 17.90	34.46 ± 13.08	[17]
SDNN	66.17 ± 16.01	56.46 ± 13.47	[17]
	52.89 ± 20.11	42.14 ± 17.87	[4]
	54.3 ± 17.0	50.7 ± 17.7	[12]

**Table 2:** Characteristics of IGD subjects based upon EEG parameters study.

**Subjects**	**EEG Parameters**	**References**
IGD and HC	Reduced no-go P3 amplitudes for the go/no-go task and blunted gain FRN amplitudes, lower no‐go N2 amplitudes in an IGD group than in the control group.	[50]
	IGD subjects have increased alpha coherence in the right hemisphere. This may lead to depressive symptoms and higher stress levels for the IGD subjects.	[52]

## Data Availability

The data and supportive information are available within the article.

## LIST OF ABBREVIATIONS

ADHD: Attention-Deficit/Hyperactivity Disorder
ANS: Autonomic Nervous System
AUDIT: Alcohol Use Disorders Identification Test
BAI: Beck Anxiety Inventory
BDI: Beck Depression Inventory
BIS-11: Barratt Impulsiveness Scale, version 11
CBCL: Child Behavior Checklist
CDI: Child Depressive Inventory
ECG: Electrocardiogram
fMRI: functional Magnetic Resonance Imaging
GMVs: Gray Matter Volumes
HF: High Frequency
HRV: Heart Rate Variability
IGD: Internet Gaming Disorder
LF: Low Frequency
ReHo: Regional Homogeneity
SUD: Substance Use Disorder
YDQ: Young Diagnostic Questionnaire

## STANDARDS OF REPORTING

PRISMA guidelines and methodology were followed.

## SUPPLEMENTARY MATERIAL

PRISMA checklist is available as supplementary material on the publisher’s website along with the published article.
